# Controlling the Ratio of Rhombohedral to Cubic Phases of In_2_O_3_ by Cobalt Doping and Its Effect on Sensor Response to Hydrogen

**DOI:** 10.3390/nano16191196

**Published:** 2026-09-22

**Authors:** Mariya I. Ikim, Varvara A. Demina, Olusegun J. Ilegbusi, Leonid I. Trakhtenberg

**Affiliations:** 1 N.N. Semenov Federal Research Center for Chemical Physics RAS, 4 Kosygin Street, Moscow 119991, Russia; ikimmary1104@gmail.com (M.I.I.); demina.varvara@yandex.ru (V.A.D.); litrakh@gmail.com (L.I.T.); 2 Department of Mechanical and Aerospace Engineering, University of Central Florida, Orlando, FL 32816, USA; 3 Moscow Center for Advanced Studies, Moscow 123592, Russia

**Keywords:** cubic/rhombohedral indium oxide, doping, cobalt ions, phase transformation, sensor response, hydrogen

## Abstract

The effect of cobalt doping in the concentration range 0.01 to 0.25 at.% on the structural and sensory properties of nanocomposites based on two-phase indium oxide synthesized by the hydrothermal method is investigated. The phase ratio is shown to change depending on the concentration of added cobalt. Co concentration of up to 0.1 at.% promotes rhombohedral-to-cubic phase transition, while concentrations exceeding 0.1 at.% stabilize the metastable phase of indium oxide. Doping leads to a reduction in particle size and an increase in the specific surface area and porosity of the composites. The sample with cobalt concentration of 0.025 at.% exhibits the maximum sensor response, which is attributed to phase transformation induced by vacancy formation. Increasing the Co concentration produces a sharp decrease in the sensor signal and a reduction in the optimal operating temperature.

## 1. Introduction

Indium oxide (In_2_O_3_) holds a special position among metal oxides used for the development of highly efficient conductometric gas sensors. In_2_O_3_ is a stable wide-bandgap n-type metal oxide semiconductor with high charge carrier mobility, and by varying the synthesis conditions, particles with diverse morphologies can be obtained [1,2,3,4]. The development of indium oxide-based composites with different metal oxides can significantly improve their gas-sensing characteristics through the formation of heterojunctions. For example, the formation of *p*–*n* and *n*–*n* heterojunctions upon the incorporation of nickel or zinc oxides, respectively, enhances the sensitivity to organic gases [5,6].

Indium oxide has two crystalline phases: a stable cubic bixbyite-type structure (c-In_2_O_3_, Ia3, with the lattice parameter a = 10.118 Å) and a metastable rhombohedral corundum-type structure (rh-In_2_O_3_, R3j, a, b = 5.478 Å and c = 14.51 Å) [7,8]. The crystalline phase type of semiconductor oxides markedly affects the concentration of oxygen vacancies, morphology at the microscopic level, and specific surface area, thereby influencing the catalytic and gas-sensitive properties of the materials [9]. All these factors make indium oxide sensitive to the presence of both reducing and oxidizing gases in the atmosphere, which explains its popularity among materials used for the fabrication of conductometric sensors [10,11,12]. Indium oxide is used not only in gas sensors but also for the development of solar cells, ultraviolet filters, photodetectors, and optoelectronic devices [13,14,15].

Currently, research on the optical, photoelectric, and sensor properties of indium oxide is primarily concentrated on the cubic oxide phase, while the rhombohedral phase has been investigated much less frequently because its synthesis requires severe conditions, including high temperature and pressure. The hydrothermal method is the main approach for obtaining pure rhombohedral indium oxide as well as phase mixtures [9,16]. The amount of rh-In_2_O_3_ in the mixture can be regulated by varying the synthesis parameters [17].

Doping can contribute to changes in both the phase composition of indium oxide and the characteristics of the crystalline phases themselves. Thus, doping with zinc or its oxide led to the transformation of cubic In_2_O_3_ into a mixture of c-In_2_O_3_ and rh-In_2_O_3_ [18]. The dopant content influences the morphology of the composites and, therefore, their sensor properties. Upon doping with zinc ions at a molar ratio of 7:1 relative to In, flower-like structures with a high specific surface area were observed, and sensors based on them exhibited higher sensitivity to NO_2_ than pure c-In_2_O_3_ [19]. Doping with Er^3+^ ions showed that their incorporation promotes the transformation of cubic In_2_O_3_ into a phase mixture and leads to an increase in photocatalytic activity [20]. Strontium doping converts cubic In_2_O_3_ into the rhombohedral phase, which leads to enhanced ethanol sensitivity [21].

Increasing the iron concentration converts pure c-In_2_O_3_ first into a mixture of c-In_2_O_3_ and rh-In_2_O_3_ phases and then into pure rh-In_2_O_3_. Furthermore, the mixed-phase indium oxide exhibited the highest response to Cl_2_ compared with pure cubic or rhombohedral In_2_O_3_ [22]. Enhanced sensitivity to acetic anhydride was observed upon the introduction of Cd into biphasic In_2_O_3_ nanospheres [23]. The incorporation of La^3+^ into In_2_O_3_ promoted changes in the size and morphology of In_2_O_3_ nanostructures, as well as stabilization of the rhombohedral phase relative to the more stable cubic polymorph [24]. The simultaneous doping of indium oxide with La and Fe increased the content of the rhombohedral phase in its nanospheres, resulting in enhanced acetone-sensing properties [25]. Thus, the introduction of various dopants into the cubic phase of indium oxide can trigger phase transformations, specifically the stabilization of the rhombohedral phase or a phase mixture, which affects various performance characteristics.

There is a dearth of information on cobalt doping accompanied by changes in the phase composition. However, the introduction of cobalt ions can significantly alter both the sensing and various structural characteristics of the host oxide. For example, Co ions partially suppressed grain growth when substituting for In in the cubic lattice [26]. The sensitivity of these sensors was considerably enhanced due to the increased concentration of oxygen vacancies. In addition, redox reactions involving cobalt ions have a positive effect on sensor efficiency. Cobalt doping of cubic indium oxide leads to a decrease in crystallite size and increase in defect concentration, which, at the optimal cobalt concentration of 0.5%, had a beneficial effect on the sensing characteristics for triethylamine detection [27]. Cobalt addition also markedly enhances the sensitivity to formaldehyde [28]. Incorporation of cobalt into both cubic and rhombohedral indium oxide leads to expansion of the crystal lattice; however, the sensor response to hydrogen decreases in the case of c-In_2_O_3_, while it increases for rh-In_2_O_3_ [29].

In this study, the effect of cobalt ion concentration on changes in the ratio between the cubic and rhombohedral phases of indium oxide was discovered and investigated using various physicochemical analysis methods. Their structural characteristics, morphology, conductivity, and sensing properties toward hydrogen were also investigated.

## 2. Materials and Methods

Two-phase indium oxide and cobalt-doped nanocomposites based on it were processed by the hydrothermal method. During the synthesis of cubic/rhombohedral In_2_O_3_, 2 mmol of In(NO_3_)_3_·5H_2_O, 18 mmol of urea, and 4 mmol of C_6_H_8_O_7_ were dissolved in 80 mL of distilled water. The resulting mixture was treated in an ultrasonic bath for 30 min at 30 °C to ensure complete dissolution of the reagents. The solution was then transferred to a 100 mL autoclave, heated to 200 °C at a rate of 4 °C/min, and maintained at this temperature for 3 h to enable the hydrothermal reaction. After cooling the autoclave to room temperature, the formed precipitate was collected by centrifugation for 15 min at 4500 rpm, washed several times with water, and subsequently dried at 90 °C. To obtain the oxide material, the precursor powders were annealed at 500 °C for 2 h. For the synthesis of the composites, a Co(NO_3_)_2_·6H_2_O solution with a concentration of 0.05 mol/L was introduced into the aforementioned reaction mixture using a micropipette in amounts of x = 0.01, 0.025, 0.1, and 0.25 at.% Co with respect to In. The synthesis procedure was then repeated. The samples obtained were designated as In_2_O_3_, 0.01CoIn, 0.025CoIn, 0.1CoIn, and 0.25CoIn.

The crystal structure of the synthesized materials was determined by XRD using a Rigaku SmartLab SE diffractometer (Rigaku, Tokyo, Japan) with Cu Kα radiation at a wavelength of 1.5406 Å. The average crystallite size was evaluated by the Debye–Scherrer equation: D = 0.9λ/(βcosθ), where λ is the X-ray radiation wavelength, β represents the full width at half maximum of the diffraction peak, and θ is the diffraction angle corresponding to that peak. The crystalline phase ratio was determined by full-profile Rietveld analysis using SmartLab Studio II software (version 4.4.241.0). JCPDS cards Nos. 71-2194, 68-0337, 16-0161, and 17-0549 were used in the calculations. The specific surface area and pore size distribution of the samples were evaluated using the low-temperature nitrogen adsorption method with a NOVA Series 1200e Quantachrome instrument (Boynton Beach, FL, USA). Particle morphology and metal ion distribution between the components of the composite were examined using SEM with a Prisma E instrument and TEM with a Tecnai Osiris FEI microscope (FEI Company, Hillsboro, OR, USA) equipped with an energy-dispersive X-ray spectroscopy (EDX) system. The electronic structure of metal ions on the nanoparticle surface in the samples was studied by XPS using a Prevac EA15 System spectrometer (PREVAC, Rogów, Poland) with Mg Kα radiation as the excitation source (1253.6 eV). The binding energy of the C 1s peak at 284.7 eV was used as a reference.

To determine the sensing properties, the synthesized materials were formed into a metal oxide film on a specialized chip [17]. For this, the nanopowders were combined with distilled water, and the resulting suspension was coated onto polycor plates equipped with a platinum heater and contacts for monitoring electrophysical characteristics. The plate with the applied suspension was heated while gradually raising the temperature to 550–600 °C and kept at this temperature until the resistance of the formed metal oxide film reached a stable value. The sensing response of the formed films upon detection of H_2_ was measured using a specially designed setup in the temperature range of 280 to 520 °C. The accuracy of temperature maintenance varied within 1 °C.

The chip with the deposited sensing layer was placed in a special chamber with a volume of approximately 1 cm^3^, into which purified air or a calibration gas mixture containing 0.9% H_2_ was supplied. The gases were passed through the chamber at a flow rate of 200 mL/min. Changes in the sensor resistance were monitored with a Keysight digital multimeter (Keysight Technologies, Santa Rosa, CA, USA), whose signal was transferred to a computer. The installed computer program generated the kinetic curve of the resistance change. The sensor response was calculated from these data using the expression S = R_0_/Rg, where R_0_ denotes the initial sensor resistance prior to the introduction of the analyzed gas mixture, and Rg is the minimum resistance value attained after exposure to the target gas.

## 3. Results and Discussions

### 3.1. Composition, Structure and Morphology

The XRD patterns of both pure indium oxide and Co-containing composites indicates reflections from the predominant cubic c-In_2_O_3_ phase (JCPDS card No. 71-2194) and the metastable rhombohedral rh-In_2_O_3_ phase (JCPDS card No. 68-0337) (Figure 1a). The XRD spectra of the intermediate products formed during hydrothermal processing contain peaks assigned to indium hydroxide In(OH)_3_ (JCPDS No. 16-0161), along with reflections corresponding to indium oxyhydroxide InOOH (JCPDS No. 17-0549) (Figure 1b).

There were no impurity phases corresponding to cobalt or its compounds observed in the XRD patterns of any of the composites or their precursors. This indicates that cobalt ions are already incorporated into the structure of indium oxide during the hydrothermal treatment stage and form a solid solution. Depending on its concentration, cobalt addition to the mixture of rh-In_2_O_3_ and c-In_2_O_3_ phases influences the phase ratio (Table 1). In particular, introducing up to 0.1 at.% Co results in a gradual increase in the content of the cubic phase from 18.5% to 20.7%. In contrast, cobalt concentration in the composite between 0.1 and 0.25 at.% leads to an increase in the fraction of the rhombohedral In_2_O_3_ phase (see Table 1).

Previous studies demonstrated that the incorporation of different ions, including Er, La, Zn, and Fe, during hydrothermal synthesis promotes the stabilization of the rhombohedral In_2_O_3_ phase with respect to the most stable cubic polymorph [20,24,25]. It is well established that the cubic modification is thermodynamically stable, while the rhombohedral phase is metastable and forms in the course of hydrothermal synthesis due to the presence of InOOH [30]. Indium oxyhydroxide is obtained through the dehydration of In(OH)_3_ formed during the hydrolysis of In^3+^ ions [31].

It has been shown that, upon the introduction of low cobalt at concentration into indium oxide, cobalt has a valence state of 2+, while at higher concentrations, a mixture of Co^2+^ and Co^3+^ ions is formed [32]. In our case, it may be suggested that at low cobalt concentrations of up to 0.1 at.%, Co ions are mainly incorporated into the cubic-phase lattice. Here, heterovalent substitution with the formation of oxygen vacancies predominates. The subsequent thermal annealing of the precursors results in acceleration of bulk diffusion, which causes an increase in the content of the cubic phase of indium oxide. As the cobalt concentration increases to 0.25 at.%, Co^3+^ ions begin to actively accumulate together with Co^2+^; and these ions have the same charge as the initial indium In^3+^. However, its substitution in the lattice does not lead to the formation of new oxygen vacancies. At the same time, local compressive stresses develop in the lattice, since the ionic radius of both Co^2+^ (0.72 Å) and Co^3+^ (0.62 Å) is smaller than that of In^3+^ (0.81 Å) [33]. To reduce the deformation, excess cobalt precipitates at the interphase boundaries of the nanoparticles, blocking atomic motion and thereby leading to an increase in the rhombohedral phase content.

Thus, low Co concentrations of up to 0.1 at.% promote the rh-In_2_O_3_ → c-In_2_O_3_ phase transition. Notably, the incorporation of high cobalt concentrations inhibits recrystallization and thus stabilizes the rhombohedral phase. This behavior results from the dopant reaching its solubility limit at lattice sites and its subsequent segregation at nanoparticle interphase boundaries. In this case, excess cobalt ions (primarily Co^3+^) impede atomic diffusion and the migration of grain boundaries. The local compressive stresses induced by the smaller ionic radius of cobalt provide an additional stabilizing effect. All these factors hinder the transformation of the rhombohedral phase into the thermodynamically stable cubic modification.

The inverse relationship between the concentrations of the final polymorphic phases of indium oxide and those of the intermediate products indicates that cobalt introduction directly regulates the dehydration and subsequent recrystallization processes of the intermediate products (see Figure 1 and Table 1). Annealing of the intermediate products leads to the formation of oxides: InOOH → rh-In_2_O_3_ and In(OH)_3_ → c-In_2_O_3_ (Figure 1). Cobalt addition at concentrations of up to 0.1 at.% promotes the dehydration of In(OH)_3_ into InOOH, resulting in an increase in the oxyhydroxide content of the intermediate products from 84.7% to 89.2% (see Table 1). At the same time, the rhombohedral phase formed from InOOH readily recrystallizes into cubic In_2_O_3_.

The incorporation of cobalt ions at these concentrations generates additional oxygen vacancies, which enhance diffusion. Therefore, although a larger amount of InOOH is formed after hydrothermal synthesis, the content of the final rhombohedral phase decreases (see Table 1). At cobalt concentrations above 0.1 at.%, cobalt inhibits the dehydration of indium hydroxide, and the fraction of InOOH in the intermediate products decreases to 82.7% (Table 1). Excess cobalt segregates at grain boundaries, thereby suppressing recrystallization. Consequently, despite the smaller initial amount of intermediate InOOH, the resulting rhombohedral phase is kinetically stabilized, preventing its transformation into the cubic phase.

Furthermore, the crystallite size for both phases is also dependent on the concentration of the incorporated cobalt (Table 1). Thus, at low cobalt concentration (0.01–0.025 at.%), the particle size of cubic indium oxide increases from 18.7 nm to 20–20.4 nm. This once again indicates accelerated atomic diffusion in the lattice due to vacancy formation, which induces crystallite growth.

Upon increasing the Co concentration, the particle size decreases, reaching 14.9 nm for 0.25CoIn as a result of dopant segregation at the boundaries. Over the entire examined range of cobalt concentrations, the size of the rhombohedral phase steadily decreases from 24 to 14 nm. The lattice parameter of both cubic and rhombohedral indium oxide also decreases in the samples with Co concentration up to 0.1 at.%, further confirming the replacement of large indium ions by smaller cobalt ions. The addition of 0.1 at.% Co leads to an expansion of the lattices of both polymorphic modifications of In_2_O_3_ (Table 1). This effect may be associated with a change in the Co^2+^/Co^3+^ ratio or with partial incorporation of cobalt into interstitial positions. In contrast, the 0.25CoIn sample exhibits a pronounced contraction of both lattices.

Nitrogen adsorption–desorption experiments were performed at 77 K to analyze the specific surface area and porosity of the synthesized samples. The adsorption–desorption isotherms of the samples, regardless of the preparation method, exhibit a type IV shape with an H3 hysteresis loop according to the IUPAC classification [34], which is characteristic of the mesoporous nature of the synthesized systems (Figure 2).

An increase in cobalt concentration leads to a monotonic rise in the BET-specific surface area from 23.3 to 25.3 m^2^/g (Table 2). The specific surface area of the composites is higher than that of pure indium oxide; therefore, cobalt doping effectively enhances the surface area. Most likely, the larger specific surface area will improve the sensor performance due to the increased number of active centers available for the adsorption of oxygen and the target gas. The BJH method was used to analyze the desorption branch of the isotherm and evaluate the mesoporous structure of the samples (inset in Figure 2, Table 2). The addition of cobalt also increases the pore volume by 27%. It should be noted that almost the entire surface area of the samples is formed by mesopores. Meanwhile, their average diameter remains virtually unchanged regardless of the cobalt concentration introduced (Table 2).

The SEM results showed that the introduction of cobalt influences the morphology of the obtained nanopowders (Figure 3). Non-doped indium oxide is characterized by nonuniform, densely packed aggregates with a wide size distribution (Figure 3a). Cobalt incorporation results in the formation of a more homogeneous, fine-grained structure with a distinct sponge-like relief (Figure 3b). These findings are consistent with the results of low-temperature nitrogen adsorption, indicatingg that the mesopore volume and specific surface area increased to 0.131 cm^3^/g and 25.3 m^2^/g, respectively, for the 0.25CoIn sample relative to pure indium oxide.

The morphology and microstructure of indium oxide and nanocomposites based on it were also studied by TEM (Figure 4). The spatial distribution of In, O, and Co was determined using EDX mapping of the corresponding elements in the composites (insets in Figure 4a,b). According to the TEM data, both pure indium oxide and the 0.25CoIn sample consist of developed aggregates formed by nanosized primary particles (Figure 4a,b). The TEM image of the sample containing 0.25 at.% cobalt shows a decrease in the average crystallite size compared with undoped oxide.

The EDX mapping analysis of In_2_O_3_ indicates a homogeneous distribution of indium and oxygen throughout the entire volume of the nanoparticles. The In and Co distribution in the composite reveals the localization of cobalt ions at interphase boundaries and nanoparticle junctions. The HRTEM image of In_2_O_3_ reveals the crystalline structure of individual nanoparticles with sizes of approximately 18–24 nm, which is consistent with the XRD data (Figure 4c and Table 1). The calculated interplanar spacing (d = 0.29 nm) corresponds to the (222) plane of the cubic phase. The orientation of the lattice planes varies within the particles, indicating the presence of two polymorphic modifications of indium oxide in the sample. The HRTEM image of 0.25CoIn also shows a decrease in nanoparticle size to 12–15 nm compared with pure oxide (Figure 4d).

The calculated interplanar spacings for most crystallites were 0.27 and 0.353 nm, corresponding to the (110) and (012) planes of rhombohedral In_2_O_3_, respectively. This also indicates the stabilization of rh-In_2_O_3_ with increasing cobalt content. It is worth noting that the pure oxide exhibits defect-free contacts between particles, while the formation of interlayers at the boundaries is observed in the 0.25CoIn sample. These interlayers are formed as a result of the anchoring of excess cobalt ions at the interphase boundaries.

XPS analysis was performed to determine the composition, valence states of the surface elements, and active oxygen centers in the samples (Figure 5). The overview spectra of pure indium oxide show characteristic In, O, and C peaks with no additional impurity elements, confirming its high purity. In composites containing cobalt, peaks of Co are recorded, the intensity of which is noticeably weaker than that of other elements due to its low content. High-resolution In 3d spectra of pure indium oxide reveal two intense peaks at binding energies of 444.13 and 451.71 eV, corresponding to In 3d5/2 and In 3d3/2, indicating the indium valence state of +3 (Figure 5a). The In 3d electron binding energy for samples containing up to 0.1 at.% remains virtually unchanged relative to In_2_O_3_. However, with increasing cobalt concentration, a clear shift in the In3d peaks toward lower binding energies of 443.94 eV is observed (Figure 5a). The shifts also indicate lattice supersaturation, cobalt incorporation into interstitial sites, and an increase in the rhombohedral phase.

High-resolution O1s XPS spectra were analyzed, as the chemical state of oxygen is of great importance for sensory performance. The asymmetric oxygen peak can be resolved into several peaks that are characteristic of three oxygen species: lattice oxygen (O_L_), oxygen vacancies (O_V_), and chemisorbed oxygen (O_C1_ and O_C2_) (Figure 5b). The O_L_ component (~529 eV) is responsible for oxygen atoms in the crystal lattice sites, Ov (~530 eV) is associated with oxygen vacancies in defective regions of the oxide, and Oc (~532–534 eV) can be attributed to various adsorbed forms of oxygen on the sample surface. For samples containing up to 0.1% cobalt, an increase in the Ov contribution to 33.30% is observed, confirming the formation of oxygen vacancies during heterovalent substitution. At the same time, at cobalt contents ≥ 0.1%, a sharp decrease in the oxygen vacancy concentration to 24.89% is detected.

### 3.2. Conductivity and Sensor Response to Hydrogen

The data obtained indicate a strong effect of cobalt additives in cubic/rhombohedral indium oxide on its phase composition, morphology, and structural characteristics. Studies of the temperature dependence of resistance and the sensing properties of the samples obtained indicate that the above-described changes also affect their performance characteristics. All samples exhibit semiconducting behavior in the temperature range 300–520 °C, implying that the resistance drops by several orders of magnitude as the temperature increases (Figure 6a). This behavior is caused by the thermal activation of electrons and their promotion from donor levels to the conduction band. At low Co concentrations of less than 0.1 at.%, the resistance decreases throughout the entire investigated temperature range, which confirms the rhombohedral-to-cubic phase transformation and the formation of oxygen vacancies. In indium oxide, these vacancies serve as electron donors; therefore, their increased concentration leads to a decrease in resistance.

The resistance of the samples increases upon Co addition starting from 0.1 at.%. Thus, the resistance of the 0.1CoIn sample is already higher than that of pure indium oxide, while at Co concentration of 0.25 at.% a sharp increase in resistance is observed. The XRD data showed that, at this concentration, the solubility limit of cobalt via substitution is most likely reached, and interstitial sites begin to be occupied. The increase in resistance indicates a decrease in electron mobility due to local distortions of the indium oxide lattice. This indicates that cobalt is located at the nanoparticle boundaries predominantly in the form of Co^3+^, forming high intercrystallite energy barriers. However, it should be noted that at high temperatures, the resistance of the 0.25CoIn sample becomes almost comparable to that of the other samples. That is, at such temperatures, the electron energy becomes sufficient to overcome these barriers.

The conductivity activation energies, E_a_, were determined from a linear fit of the ln(1/R) versus 1000/T dependences. The values obtained were 0.34 eV for In_2_O_3_ and 0.31, 0.28, 0.37, and 0.91 eV for the samples with Co contents of 0.01, 0.025, 0.1, and 0.25 at.%, respectively. Analysis of the E_a_ values indicates their correlation with the effect of the introduced cobalt concentration on the structure of the samples according to the XRD and XPS data (Figure 1 and Figure 5, Table 1). With cobalt concentrations up to 0.1%, a decrease in the activation energy to a minimum value of 0.28 eV is observed. This effect is directly associated with the heterovalent incorporation of Co^2+^ ions into the lattice sites of the cubic indium oxide phase, which induces the generation of additional donor centers that supply electrons to the conduction band. At 0.1 at.% Co, E_a_ increases to 0.37 eV, which is associated with cobalt occupying interstitial positions. For the 0.25Co sample, a sharp increase in the activation energy to 0.91 eV is observed, indicating that electron transport at this doping level requires overcoming a high activation barrier.

The behavior of the temperature-dependent resistance curves shown in Figure 6a and the conductivity activation energies is related to the structural and phase changes revealed by XRD and XPS. The incorporation of cobalt influences the kinetics of dehydration and subsequent recrystallization, which directly affects the electrophysical properties of the samples. In the cobalt concentration range up to 0.1 at.%, hydrothermal conditions facilitate the dehydration of In(OH)_3_ into InOOH, whose fraction reaches 89.2% (see Table 1). Upon subsequent annealing, InOOH converts into the metastable rhombohedral phase of In_2_O_3_.

However, heterovalent doping induces an excess concentration of oxygen vacancies, as evidenced by the XPS data. The resulting vacancies decrease the diffusion barrier and facilitate bulk recrystallization, leading to an intensive transformation of the metastable phase into the thermodynamically stable cubic In_2_O_3_ phase. In this case, electrons donated by oxygen vacancies are intensively injected into the conduction band, causing the resistance to decrease over the entire temperature range and the activation energy for conduction to reach its minimum value. It is the rapid recrystallization and generation of charge carriers that account for the lowest resistance of the samples with cobalt composition of up to 0.1 at.%.

At cobalt concentrations above 0.1 at.%, the opposite trend in the phase composition is observed (see Table 1). During hydrothermal synthesis, cobalt begins to inhibit dehydration, causing the InOOH fraction to decrease to 82.7%. However, during annealing, excess cobalt ions, predominantly in the isovalent state, no longer generate oxygen vacancies. In addition, the XPS data indicates that the fraction of oxygen vacancies decreases to 24.89%. Cobalt forms amorphous interlayers that hinder atomic diffusion and suppress grain-boundary migration. As a result, the metastable rhombohedral phase is prevented from transforming into the cubic modification.

The suppression of recrystallization and the loss of oxygen vacancies lead to a decrease in the electron concentration in the conduction band. Simultaneously, high intergranular potential barriers develop at the grain boundaries. As a result, the baseline resistance increases by several orders of magnitude (see Figure 6a), and the activation energy for electrical conduction reaches 0.91 eV. Electron transport in this system can therefore occur only at elevated temperatures, at which the electrons acquire sufficient thermal energy to overcome the cobalt-induced grain-boundary barriers.

A maximum is observed in the temperature-dependent sensor response during the detection of 0.9% H_2_, and its position varies with temperature (Figure 6b). Such a maximum is typical of semiconductor sensors and is governed by the competition between two processes: the acceleration of surface chemical reactions between hydrogen and chemisorbed oxygen, and gas desorption at excessive heating. For the samples containing up to 0.1 at.% Co, the operating temperature remains nearly unchanged at approximately 440 °C (Figure 6b). However, for the 0.25Co sample, a sharp shift in the maximum to 340 °C is observed. Most likely, this is associated with a size effect: at low cobalt additions, the particle size remains almost unchanged, while at a higher Co concentration, it decreases sharply (see Table 1 and Table 2). Hydrogen reaction on such a highly developed surface proceeds much more readily and rapidly; therefore, the sensor response is observed at a lower temperature. The signal amplitude also depends on the cobalt concentration.

The introduction of small amounts of cobalt leads to an increase in the sensor response to hydrogen. Thus, for the sample with a Co content of 0.01 at.%, the R_0_/R_g_ ratio increases to ~115. The maximum sensor response was demonstrated by the 0.025 at.% Co sample, where R_0_/R_g_ reaches a value of ~124. According to the XPS data, heterovalent substitution of In^3+^ by Co^2+^ ions in the bulk of the cubic-phase lattice leads to the formation of the maximum amount of surface oxygen vacancies (Figure 5). These vacancies serve as donor centers and provide active sites for oxygen chemisorption. As a result, the subsequent sensing reaction with hydrogen is accelerated. The large sensor response in experiments with the 0.025 at.% Co sample is also promoted by the developed specific surface area (24.8 m^2^/g) and mesopore volume (0.111 cm^3^/g), which facilitates diffusion and access of hydrogen molecules to the sensing layer.

Further increasing the cobalt concentration leads to a deterioration of the sensing properties. For the 0.1 at.% Co sample, the maximum response decreases to ~93, which is most likely caused by reaching the solubility limit of cobalt at lattice sites, its partial migration into interstitial positions, and the accompanying decrease in oxygen vacancies. A sharp decrease in sensitivity is observed for the 0.25 at.% Co sample (Figure 6b). According to TEM data, excess cobalt ions, predominantly Co^3+^, are expelled from the grain bulk to the nanoparticle boundaries, reducing the oxygen vacancy fraction to 24.89% according to XPS data (see Figure 4 and Figure 5). Cobalt ions formed at the crystallite boundaries limit the nanoparticle size to 14 nm and increase the total porosity to its maximum value (see Table 1 and Table 2). This promotes cobalt-induced blocking of individual particles, resulting in an increase in the activation energy of conductivity to 0.9 eV. The advantage of the maximum surface area and well-developed pore volume of the 0.25CoIn sample is completely offset by the effect of electronic blocking, which leads to a decrease in the sensor signal.

Hydrothermally synthesized undoped indium oxide constitutes a biphasic system comprising cubic and rhombohedral polymorphs. The electron work function of the metastable rhombohedral phase (W_rh-In2O3_ = 4.3 эB) is lower than that of the thermodynamically stable cubic phase (W_c-In2O3_ = 5.0 эB) [35]. Upon direct contact between the two polymorphs, a polymorphic heterojunction is formed. Due to the difference in their work functions, electrons are transferred from rh-In_2_O_3_ to c-In_2_O_3_ until Fermi-level equilibration is reached. The resulting charge redistribution induces band bending near the phase boundary, thereby promoting spatial charge separation and giving rise to an interfacial potential barrier.

The introduction of cobalt ions provides an effective means of electronic and phase modulation of this barrier and alters the electrophysical properties of the sensing layer. The incorporation of Co^2+^ ions is accompanied by the formation of donor defects, namely oxygen vacancies. Concurrently, the Fermi level shifts closer to the conduction band minimum. Electron transport is facilitated in the samples containing up to 0.1 at.% Co, which agrees with their minimum baseline resistance. At higher doping levels, cobalt accumulates predominantly in the Co^3+^ state and segregates at interphase boundaries, where it acts as deep electron traps. As a result, the Fermi level shifts downward, while the magnitude of band bending and the height of the inter-grain potential barriers increase sharply. Accordingly, the activation energy for electrical conduction increases markedly for the 0.25CoIn sample. Electron transport is hindered by the high potential barriers formed at the grain boundaries. Thus, cobalt doping enables the modulation of the potential-barrier parameters at phase interfaces, providing an optimal balance between the electrical conductivity of the material and its reactivity toward the target gas.

Selectivity, response time, reproducibility, and long-term stability are important characteristics that determine the practical applicability of gas sensors. Figure 6c shows the sensor response values of the synthesized materials upon exposure to 0.9% H_2_, CO, and NH_3_. All samples exhibit pronounced selectivity toward hydrogen. It should be noted that cobalt incorporation not only increases the sensor response to hydrogen but also suppresses the sensitivity to interfering gases. The highest selectivity coefficients toward CO and NH_3_, reaching 6.3 and 8.0, respectively, were obtained for the 0.025CoIn sample.

To evaluate long-term stability, the hydrogen response and baseline resistance of the obtained samples were monitored over a period of two months. All synthesized materials demonstrated high stability, with variations in the measured parameters remaining below 5% throughout the testing period. Figure 6d shows the dynamic response and recovery curves of the sensors based on pristine In_2_O_3_ and the sample containing 0.025 at.% Co upon exposure to and subsequent removal of 0.9% H_2_. The response time, defined as the time required to reach 90% of the maximum change in resistance, was less than 1 s for both samples. The resistance returned to its initial value after the hydrogen supply was stopped. The recovery time was approximately 5 s, indicating rapid desorption of the sensing-reaction products from the material surface and excellent process reversibility. Thus, doping with 0.025 at.% Co preserves the rapid sensor response while simultaneously increasing the magnitude of the sensing signal.

## 4. Conclusions

The effect of the concentration of introduced cobalt from 0.01 to 0.25 at.% on the composition, structure, morphology, conductivity, and hydrogen sensor response of cubic/rhombohedral indium oxide was investigated. Small cobalt additions up to 0.1 at.% promote the rh-In_2_O_3_ → c-In_2_O_3_ phase transition. At concentrations of 0.1–0.25 at.%, cobalt stabilizes the rhombohedral phase. The introduction of cobalt leads to the formation of a homogeneous finely dispersed structure with a developed spongy relief. The specific surface area monotonically increases from 23.3 to 25.3 m^2^/g, and the mesopore volume rises by 27%. Under low-level doping, Co^2+^ ions replace In^3+^, resulting in the formation of the maximum number of oxygen vacancies. At higher concentrations, cobalt accumulates in the form of Co^3+^ ions, while the fraction of oxygen vacancies decreases.

Doping up to 0.1 at.% Co decreases the resistance and the activation energy of conductivity due to the generation of donor vacancies. At 0.25 at.% Co, a sharp increase in resistance and a rise in Ea are observed due to the formation of intercrystallite energy barriers by cobalt ions at grain boundaries. The 0.025CoIn sample was found to be the optimal composition, demonstrating the highest hydrogen sensor response of 124 at an operating temperature of 440 °C. This behavior is caused by the highest concentration of surface oxygen vacancies and a developed porous structure. For the sample with 0.25 at.% Co, a pronounced size effect was found: the sharp reduction in particle size lowered the operating temperature to 340 °C, while the sensor signal amplitude decreased due to hindered electron transport.

It has been established that the introduction of cobalt ions into a mixture of cubic/rhombohedral indium oxide makes it possible to purposefully regulate the phase composition, structure, and morphology. This, in turn, leads to changes in the conductivity and hydrogen sensing properties of the rh/c-In_2_O_3_ mixture.

## Figures and Tables

**Figure 1:**
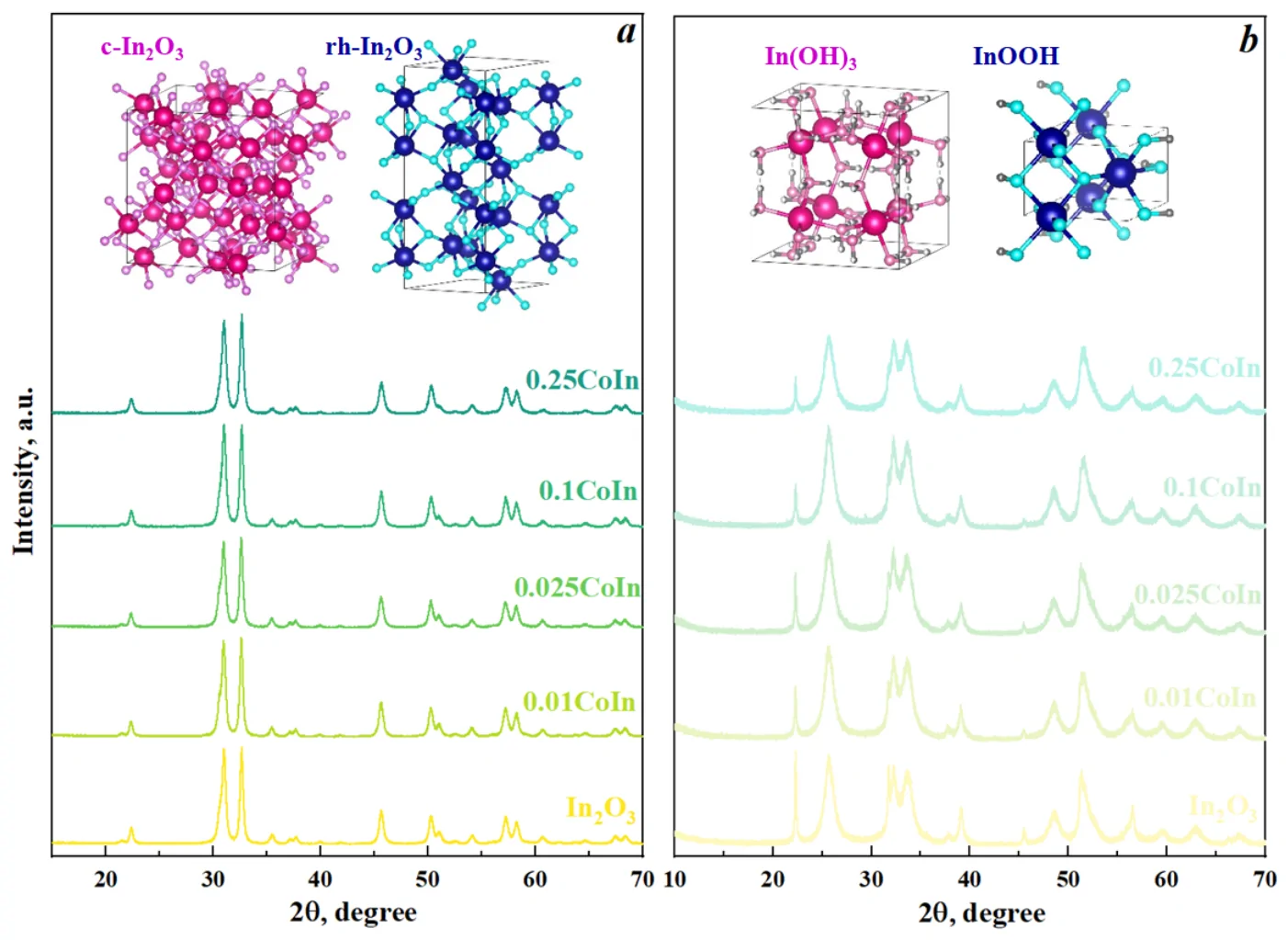
X-ray spectra of In_2_O_3_ and CoIn samples obtained by annealing (**a**), and intermediate products obtained by hydrothermal treatment (**b**). The insets show the atomic crystal structures of c-In_2_O_3_, rh-In_2_O_3_, In(OH)_3_, and InOOH.

**Figure 2:**
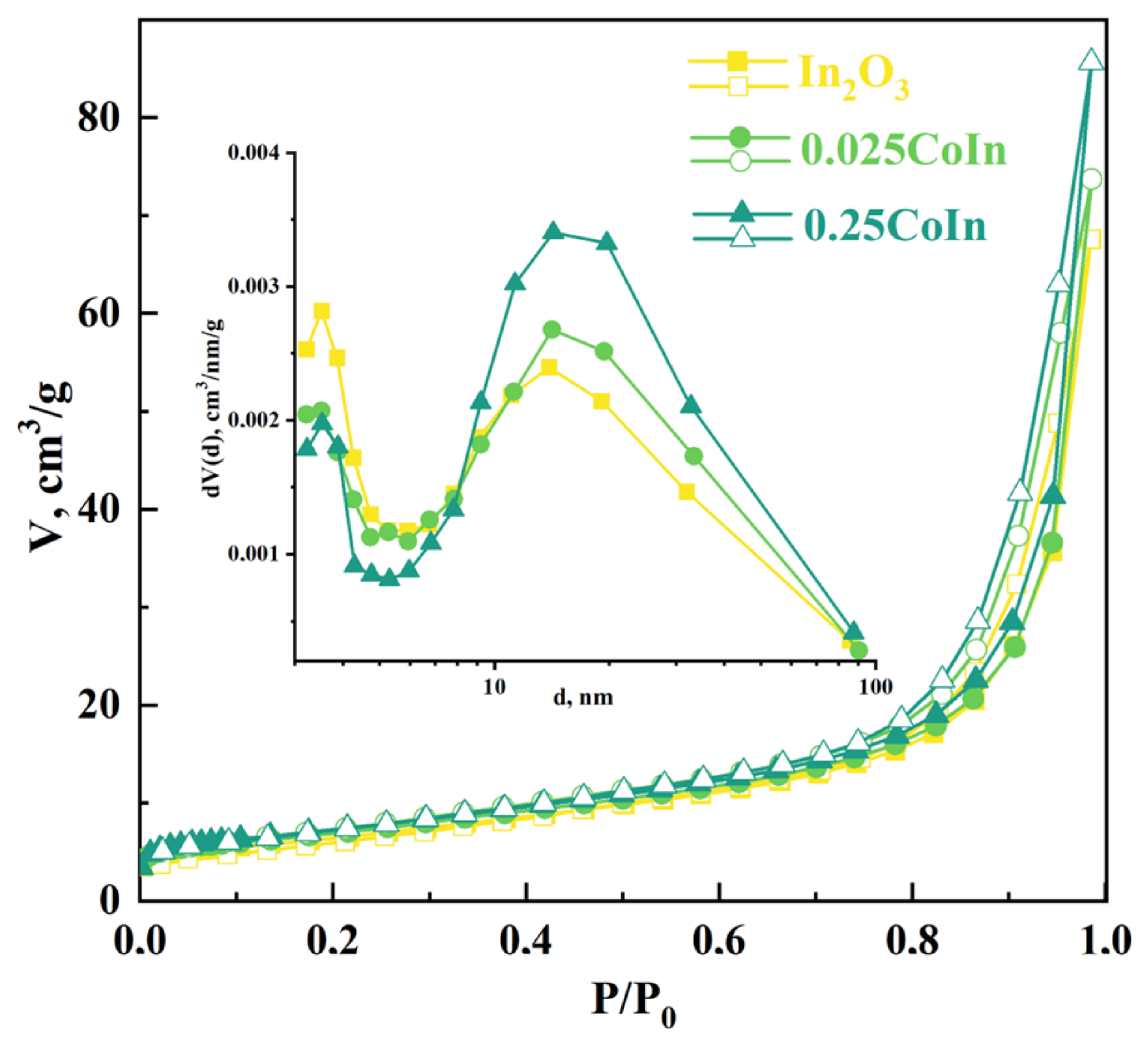
Adsorption (filled symbols) and desorption (empty symbols) isotherms of N_2_ at a temperature of 77 K for In_2_O_3_, 0.025CoIn, 0.25CoIn samples. The inset shows the distribution of pore sizes.

**Figure 3:**
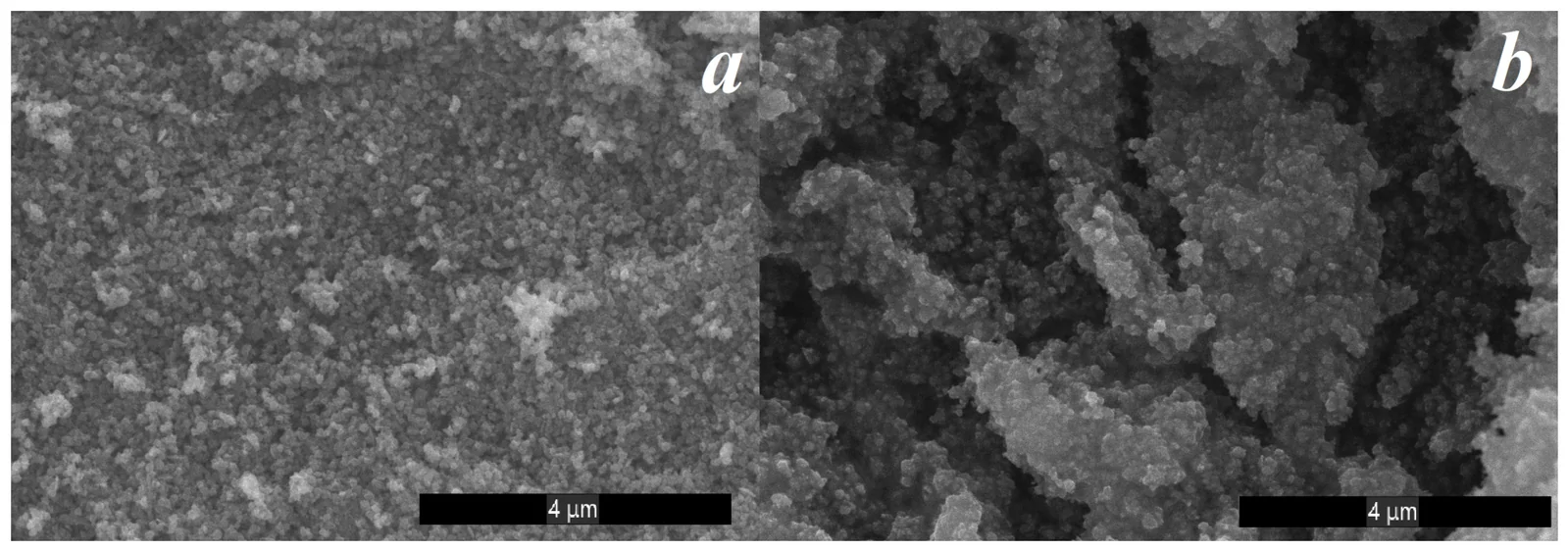
SEM images of In_2_O_3_ (**a**) and sample 0.25CoIn (**b**).

**Figure 4:**
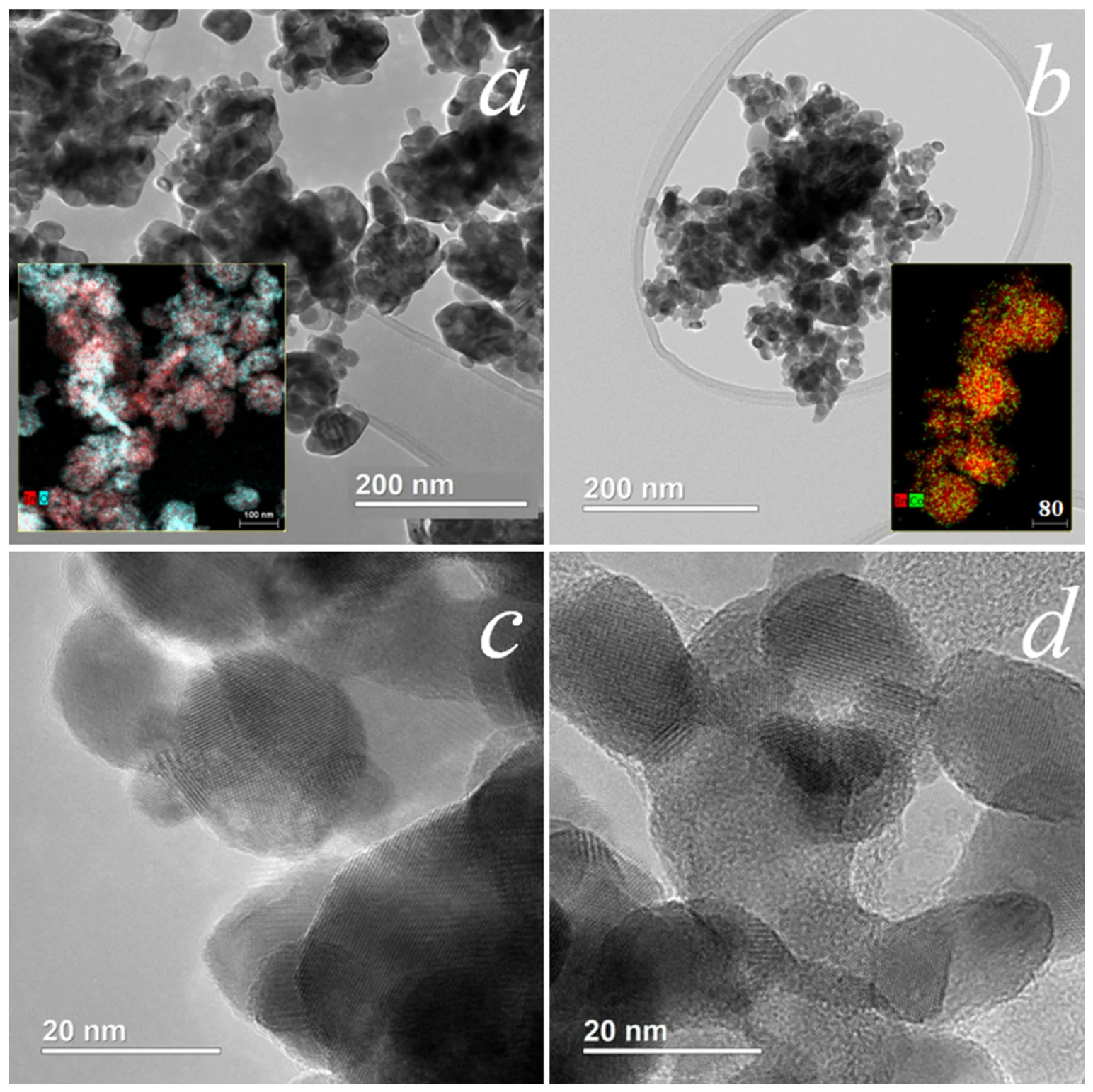
TEM—images of indium oxide (**a**) and 0.25CoIn (**b**), with EDX mapping of In, O and Co in the insets; HRTEM—images of indium oxide (**c**) and 0.25CoIn (**d**).

**Figure 5:**
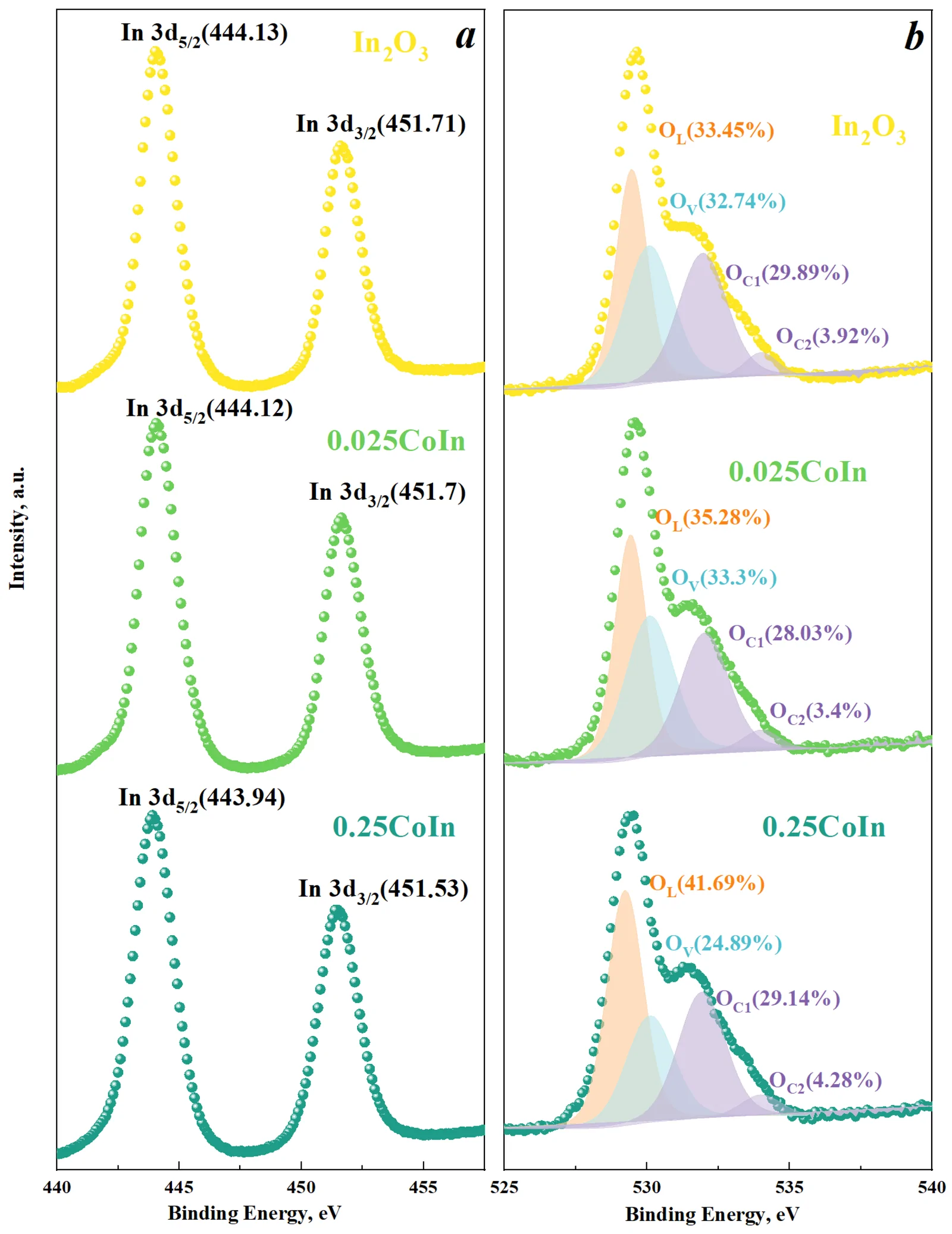
XPS spectra: In 3d (**a**) and O1s (**b**) for In_2_O_3_, 0.025CoIn, and 0.25CoIn samples.

**Figure 6:**
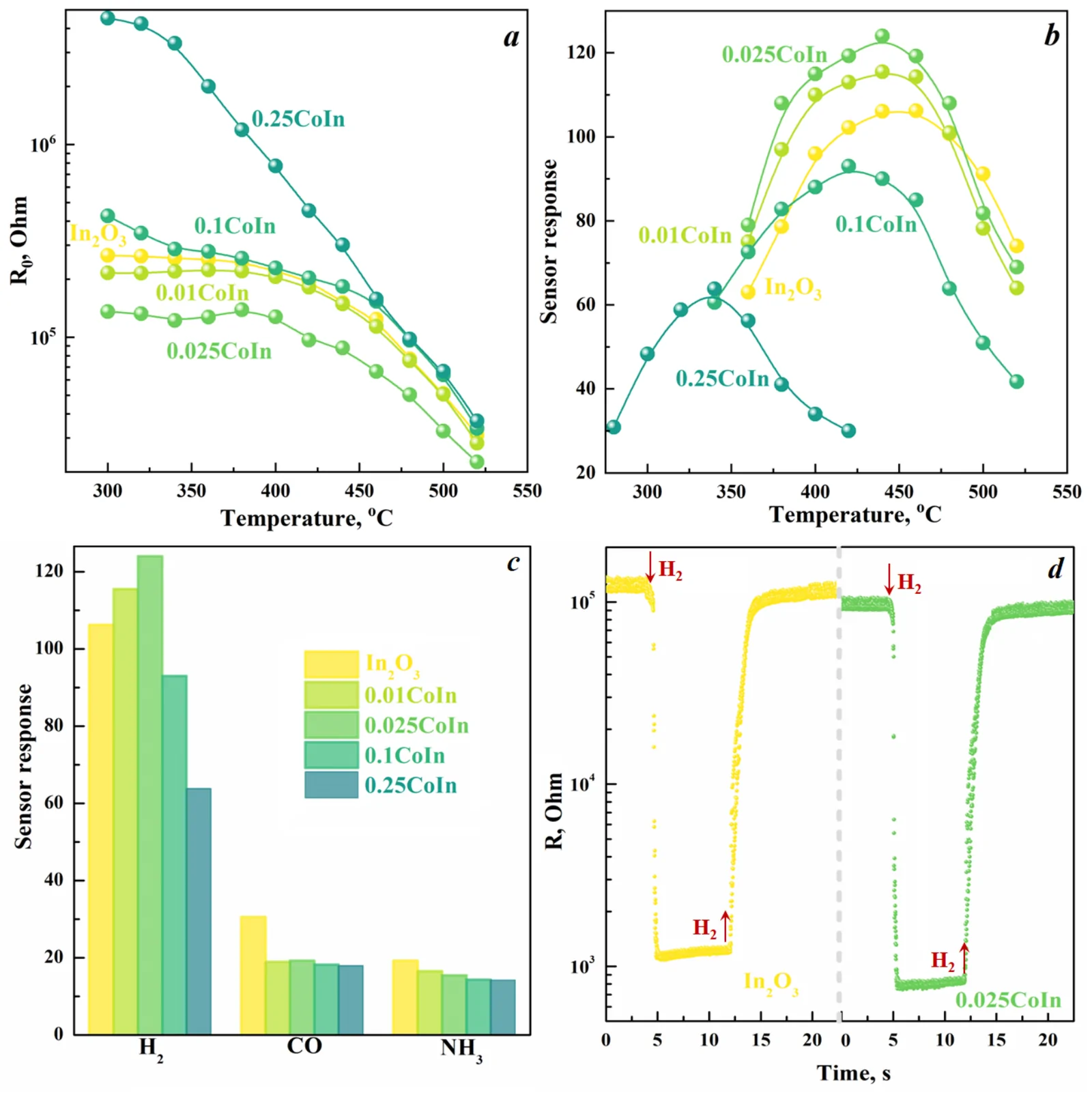
Temperature dependences of the resistance (**a**) and sensor response to 0.9% H_2_ (**b**) of In_2_O_3_ and CoIn samples. Selectivity of the samples to 0.9% H_2_, CO, and NH_3_ (**c**). Dynamic response–recovery curves for In_2_O_3_ and 0.025CoIn (**d**).

**Table 1:** XRD data for In_2_O_3_ and composites with different cobalt contents.

Sample	Weight Fraction, wt.%		Particle Size, nm		Lattice Parameter, Å	
	rh-In_2_O_3_/InOOH	c-In_2_O_3_/In(OH)_3_	rh-In_2_O_3_	c-In_2_O_3_	a = b; c	a = b = c
In_2_O_3_	81.5/84.7	18.5/15.3	24	18.7	5.48239; 14.50348	10.11675
0.01CoIn	79.8/89.2	20.2/10.8	23.3	20.4	5.48277; 14.50321	10.11660
0.025CoIn	79.3/88.5	20.7/11.5	22.5	20	5.47926; 14.49321	10.10567
0.1CoIn	82.7/84.7	17.3/15.3	22	18.5	5.48650; 14.51388	10.12141
0.25CoIn	88/82.7	12/17.3	14	14.9	5.47637; 14.48958	10.10331

**Table 2:** The parameters of mesoporous structure of the samples.

Sample	S_BET_, m^2^/g	V_mes_, cm^3^/g	S_mes_, m^2^/g	d_mes_, nm
In_2_O_3_	23.3	0.103	21.2	13.9
0.01CoIn	24.1	0.109	21.4	14
0.025CoIn	24.8	0.111	21.8	14.1
0.1CoIn	25	0.126	23.4	14.1
0.25CoIn	25.3	0.131	24.0	14.2

## Data Availability

Data is contained within the article.

## References

[1] Sugumaran S. (2026). Indium Oxide (In_2_O_3_) Gas Sensors-An Insight Study. Micro Nanostructures.

[2] Morulane K.L., Swart H.C., Motaung D.E. (2024). A review on topical advancement and challenges of indium oxide based gas sensors: Future outlooks. J. Environ. Chem. Eng..

[3] Shah S., Hussain S., Din S.T.U., Shahid A., Amu-Darko J.N.O., Wang M., Tianyan Y., Liu G., Qiao G. (2024). A review on In_2_O_3_ nanostructures for gas sensing applications. J. Environ. Chem. Eng..

[4] Yang S., Yin H., Wang Z., Lei G., Xu H., Lan Z., Gu H. (2023). Gas sensing performance of In_2_O_3_ nanostructures: A mini review. Front. Chem..

[5] Wang G., Wang Y., Guo L., Zhang G., Duan R., Wang J., Liu X., Yang Y., Yang X. (2025). Enhancing n-butanol gas sensing via NiO@ In_2_O_3_ pn junctions in magnetron-sputtered hollow-sphere-array thin films. Sens. Actuators B Chem..

[6] Zhang G., Wang Y., Ma Z., Zhang J., Lei Y., Zhai Y., Wang J., Liu X., Wang G., Guo L. (2026). An optimized ppb-level n-butanol gas sensor: ZnO@ In_2_O_3_ nn heterojunction hollow sphere array film. Sens. Actuators B Chem..

[7] Bekheet M.F., Schwarz M.R., Kroll P., Gurlo A. (2015). Kinetic control in the synthesis of metastable polymorphs: Bixbyite-to-Rh_2_O_3_ (II)-to-corundum transition in In_2_O_3_. J. Solid State Chem..

[8] Cao E., Wu L., Zhang Y., Sun L., Yu Z., Nie Z. (2023). Hydrothermal synthesis of cubic-rhombohedral-In_2_O_3_ microspheres with superior acetone sensing performance. Appl. Surf. Sci..

[9] Gulevich D., Weimar U., Bârsan N. (2026). Crystal structure, electronic properties and surface chemistry of In_2_O_3_ polymorphs as a basis for gas sensors application: A comparative analysis with SnO_2_ and WO_3_. Sens. Actuators B Chem..

[10] Tian M., Wu Y., Zhao Z., Chen Z., Zhu N., Jiang X. (2026). Materials design strategies for semiconducting metal-oxide chemiresistive gas sensors: A review. RSC Appl. Interfaces.

[11] Zhao N., Cui Y., Chang X., Zheng W., Liu X., Zhang J. (2026). Low-Dimensional In_2_O_3_ Nanostructures for Advanced Room-Temperature NO2 Sensors. Sens. Actuators B Chem..

[12] Liu Z., Jia J., Zhao G. (2026). Recent advances in modified In_2_O_3_-based gas sensors for ultra-sensitive formaldehyde detection. Talanta.

[13] Yap B.K., Zhang Z., Thien G.S.H., Chan K.Y., Tan C.Y. (2023). Recent advances of In_2_O_3_-based thin-film transistors: A review. Appl. Surf. Sci. Adv..

[14] Li J., Qu Y., Liu Y., Jing L. (2024). Design, modulation and current advances in energy and environmental applications over In_2_O_3_-based photocatalysts. Sep. Purif. Technol..

[15] Dhamodaran M., Yadav R.K., Gupta R. (2025). Ferromagnetism in In_2_O_3_-based nanostructures: A review on structure, shape, electronic structure, magnetic properties, DFT modeling and applications. J. Magn. Magn. Mater..

[16] Jin Z., Mou Y., Zhao J., Chen C., Zhou H., Xiang N., Wang F., Wang Z., Liu J., Wu L. (2024). Crystalline structure controlled of In_2_O_3_ sensing platforms for sensitive H2S performance at low temperature. Sens. Actuators B Chem..

[17] Ikim M.I., Demina V.A., Spiridonova E.Y., Ilegbusi O.J., Trakhtenberg L.I. (2025). Structural and Gas-Sensitive Characteristics of In_2_O_3_: Effect of Hydrothermal/Solvothermal Synthesis Conditions. Micromachines.

[18] Lin C.Y., Fang Y.Y., Lin C.W., Tunney J.J., Ho K.C. (2010). Fabrication of NO_x_ gas sensors using In_2_O_3_–ZnO composite films. Sens. Actuators B Chem..

[19] Li P., Fan H., Cai Y., Xu M., Long C., Li M., Lei S., Zou X. (2014). Phase transformation (cubic to rhombohedral): The effect on the NO2 sensing performance of Zn-doped flower-like In_2_O_3_ structures. RSC Adv..

[20] Lemos S.C., Romeiro F.C., de Paula L.F., Gonçalves R.F., de Moura A.P., Ferrer M.M., Longo E., Patrocinio A.O.T., Lima R.C. (2017). Effect of Er^3+^ ions on the phase formation and properties of In_2_O_3_ nanostructures crystallized upon microwave heating. J. Solid State Chem..

[21] Song L., Dou K., Wang R., Leng P., Luo L., Xi Y., Kaun C.-C., Han N., Wang F., Chen Y. (2020). Sr-doped cubic In_2_O_3_/rhombohedral In_2_O_3_ homojunction nanowires for highly sensitive and selective breath ethanol sensing: Experiment and DFT simulation studies. ACS Appl. Mater. Interfaces.

[22] Li P., Cai C., Cheng T., Huang Y. (2017). Hydrothermal synthesis and Cl_2_ sensing performance of porous-sheets-like In_2_O_3_ structures with phase transformation. RSC Adv..

[23] Zhang H., Guo Y., Zhang H., Zhu H., Meng F. (2024). Cd-Doped Biphase In_2_O_3_ Nanospheres with High Sensitivity and Selectivity for Acetic Anhydride Sensors. ACS Appl. Nano Mater..

[24] Lemos S.C.S., Nossol E., Ferrari J.L., Gomes E.O., Andres J., Gracia L., Sorribes I., Lima R.C. (2019). Joint theoretical and experimental study on the La doping process in In_2_O_3_: Phase transition and electrocatalytic activity. Inorg. Chem..

[25] Wu L., Cao E., Zhang Y., Sun L., Sun B., Yu Z. (2023). La and Fe co-doped walnut-like cubic-rhombohedral-In_2_O_3_ for highly sensitive and selective detection of acetone vapor. Mater. Lett..

[26] Yang Z., Chen X., Chen Q., Qu J., Guo Y., Zhou K., Wang T., Dastan D., Wang X., Wang F. (2025). Co ions doping enhances n-butanol sensing performance of In_2_O_3_ nanospheres. Sens. Actuators B Chem..

[27] Wang X., Li Y., Jin X., Sun G., Cao J., Wang Y. (2024). The effects of Co doping on the gas sensing performance of In_2_O_3_ porous nanospheres. Sens. Actuators B Chem..

[28] Lin G., Chen Y., Lei Q., Feng Q., Liang Q., Li G.D. (2025). Modulating surface chemisorbed oxygen of cobalt-doped In_2_O_3_ microspheres for ppb-level formaldehyde detection. J. Alloys Compd..

[29] Ikim M.I., Gerasimov G.N., Erofeeva A.R., Gromov V.F., Ilegbusi O.J., Trakhtenberg L.I. (2024). Cobalt doped cubic and rhombohedral In_2_O_3_: The role of crystalline phase of indium oxide in sensor response to hydrogen. Chem. Phys. Lett..

[30] Jiang X.B., Jiang M., Zhao M. (2013). Thermodynamic understanding of phase transitions of In_2_O_3_ nanocrystals. Chem. Phys. Lett..

[31] Song P.Y., Zhang W.D. (2014). Controlled synthesis and gas sensing properties of In_2_O_3_ with different phases from urchin-like InOOH microspheres. Mater. Res. Bull..

[32] Kumagai H., Hara Y., Sato K. (2019). Site occupancy, valence state, and spin state of Co ions in Co-doped In_2_O_3_ diluted magnetic semiconductor. J. Magn. Magn. Mater..

[33] Meng X., Tang L., Li J. (2010). Room-temperature ferromagnetism in Co-doped In_2_O_3_ nanocrystals. J. Phys. Chem. C.

[34] Brunauer S., Emmett P.H., Teller E. (1938). Adsorption of gases in multimolecular layers. J. Am. Chem. Soc..

[35] Chen F., Yang M., Wang X., Song Y., Guo L., Xie N., Kou X., Xu X., Sun Y., Lu G. (2019). Template-free synthesis of cubic-rhombohedral-In_2_O_3_ flower for ppb level acetone detection. Sens. Actuators B Chem..

